# Notices

**Published:** 1864-07

**Authors:** 


### NOTICES.

THE American Tract Society, No. 150 Nassau street, New-York, is issuing several volumes exceedingly appropriate to the times, as their several titles will indicate: "United States Primer," eighty-four pages, 12mo. "Advice to Freedmen," by Rev. J. W. Brinkerhoff, sixty-four pages, paper cover, closing with fourteen beautiful hymns. Tens of thousands of both these little volumes should be circulated. "I wish I was Poor," a story for little girls. "The Weed with an Ill Name" is a most instructive and suggestive little book for all classes of persons; it is full of life-lessons, and wisest are they who learn them earliest. "Soldiers and Soldiers' Homes," a vest-pocket book by Mrs. Phelps. We earnestly wish every one of our soldiers could be presented with one. "The Anglo-American Sabbath," by the Rev. Philip Schaff, D.D., read before the National Convention, at Saratoga, August 11th, 1863. Where the holy Sabbath is best observed, there are the best, the most law-abiding individuals in all climes; the happiest families, the most prosperous communities, and the greatest and noblest nations of all ages; and they who labor for the better observance of the Sabbath-day, with the greatest intellects, do more for a nation's radical good than the greatest generals.

Business Success.—The Messrs. Scott & Baldwin, No. 505 Broadway, New-York, owe their success to the steady observance of a few general business principles, which, if carried out uniformly among mercantile and other buyers and sellers, would insure success in almost every case, and would prevent an amount of human sorrow and destitution which no array of figures could intelligibly express to the human understanding; it would shut the door against falsehood and other human meannesses, which in amount would be utterly incalculable; and, if this article should lead a tithe of our readers to resolve upon the adoption of the business methods about to be stated, we shall consider that we have lived to no small purpose in our day and generation.

Many years ago the gentlemen above referred to began business together, and so have amicably and pleasurably continued to the present hour, never having made even an approach to a "failure," never having had occasion, in any of the commercial crises which have, like a ruthless tornado, swept thousands of our most active and wary merchants into hopeless bankruptcy, been under the necessity of asking even "an extension."

1. They began with the inflexible determination never to purchase what they could not conveniently pay for on the spot.

2. Never to ask or give credit for any article purchased.

3. Never to abate a penny on any article offered for sale.

4. Never to sell an article which was not, in all respects, what they claimed it to be.

5. Never to give short change or of a kind inferior to the note offered.

6. Never to allow a clerk to remain an hour who should exhibit the slightest discourtesy or want of patience to any customer for any cause.

Here are six business rules which are worthy of being committed to memory by every youth in the nation who has an ambition to become a successful business man.

We are sure that any of our readers who have occasion to deal with the gentlemen above named will find it both pleasurable and profitable.

"The People's Journal of Health" is a comely-looking monthly from Chicago, Illinois, at one dollar a year, edited by Drs. Hayes and Blackall. We welcome it as a co-laborer with us in the great cause of instructing the people as to the best method of keeping well. This third number of Vol. I., the first which reaches us, abounds in good things, written wisely and well. We wish every State could have its own Journal of Health, ably conducted; the tendency would be to promote a taste for this kind of reading, with the inevitable effect of largely increasing our own subscription-list. We believe that there is a plenty of room on this "mundane sphere" for every body; for every brother, laboring in a good cause, to flourish and prosper.

We are glad to notice The American Artisan and Patent Record, published weekly, at two dollars a year, by Brown, Coombs & Co., No. 212 Broadway, New-York. Mr. Brown was in the office of our particular pet, the *Scientific American*, for many years, and helped to make that unequaled paper the great success it is. We hope that the *Artisan* will merit and attain the success and high character of the *Scientific* without the least prejudice to the latter; but if Mr. Brown ever gets in sight or hailing distance of Mr. Munn, he will have to keep wide awake, for the "Scientific" has the start, has the inside track, and a bottom which can't be knocked out!

American Tract Society, Boston, No. 28 Cornhill, and No. 13 Bible House, New-York, have issued "New Stories from an old Book," 216 pages, with illustrations. "Walter Lightfoot's Pictures," 180 pages, by Mrs. H. E. Brown. "The Gospel among the Caffres, or the Story of Rev. Mr. Moffat, and his Labors in South-Africa," 284 pages. "Ancient Egypt, its Antiquities, Religion, and History to the Close of the Old Testament Period," by the Rev. George Trevor, M.A., Canon of York, 400 pages. The first two volumes are specially adapted to secure the attention of children, and to inculcate sound, practical principles; the last two are of very great value as veritable histories, and would be important additions to any library, public or private.

The whole country owes a very great deal to the energy, enterprise, and judgment of the managers of the Boston American Tract Society, and the money spent at its rooms is not only a personal benefit to the purchaser, but places means in the hands of the Society of extending its beneficent operations to all lands.

"The Union Monthly and Journal of Health and Education," a dollar monthly, Philadelphia, Pa., edited by Wm. M. Cornell, M.D., LL.D. Dr. Cornell is a veteran in the science and practice of medicine. The public are familiar with his contributions to the press, which are uniformly sound in principle, safe in practice, and always of a high moral tone. We wish him a great success.

To Farmers. — That very excellent agricultural weekly, "The Country Gentleman," begins a new volume with July, $2.50 a year, Albany, N. Y. It has been conducted with great ability for many years, and any farmer's family can not fail to be benefited many times its subscription price, if it is carefully and habitually read, and its suggestions as to the most certain and profitable modes of managing all that pertains to the farm are attended to. It is a most miserable economy not to take some agricultural publication. Certainly no farmer of intelligence, thrift, and influence would do without one, at five times the present cost.

